# Study of Thermometry in Two-Dimensional Sb_2_Te_3_ from Temperature-Dependent Raman Spectroscopy

**DOI:** 10.1186/s11671-020-03463-1

**Published:** 2021-02-03

**Authors:** Manavendra P. Singh, Manab Mandal, K. Sethupathi, M. S. Ramachandra Rao, Pramoda K. Nayak

**Affiliations:** 1 Department of Physics and Materials Science Research Centre, Indian Institute of Technology Madras, Chennai, 600 036 India; 2 Department of Physics, Indian Institute of Technology Madras, Chennai, 600 036 India; 3 Nano Functional Materials Technology Centre, Indian Institute of Technology Madras, Chennai, 600 036 India

**Keywords:** Thermometry, Topological insulators, Thermoelectric, Micro-Raman, Figure of merit, Thermal conductivity

## Abstract

Discovery of two-dimensional (2D) topological insulators (TIs) demonstrates tremendous potential in the field of thermoelectric since the last decade. Here, we have synthesized 2D TI, Sb_2_Te_3_ of various thicknesses in the range 65–400 nm using mechanical exfoliation and studied temperature coefficient in the range 100–300 K using micro-Raman spectroscopy. The temperature dependence of the peak position and line width of phonon modes have been analyzed to determine the temperature coefficient, which is found to be in the order of 10^–2^ cm^−1^/K, and it decreases with a decrease in Sb_2_Te_3_ thickness. Such low-temperature coefficient would favor to achieve a high figure of merit (*ZT*) and pave the way to use this material as an excellent candidate for thermoelectric materials. We have estimated the thermal conductivity of Sb_2_Te_3_ flake with the thickness of 115 nm supported on 300-nm SiO_2_/Si substrate which is found to be ~ 10 W/m–K. The slightly higher thermal conductivity value suggests that the supporting substrate significantly affects the heat dissipation of the Sb_2_Te_3_ flake.

## Introduction

Topological insulators (TIs) are the new class of quantum materials having a wide energy gap in the bulk and surface gap less Dirac-like states, which are protected under time-reversal symmetry [1–3]. These materials hold great promise for a broad range of potential applications, including field-effect transistors [4, 5], infrared-THz detectors [6], magnetic field sensors [7, 8] and thermoelectricity [9, 10]. The thermoelectric performance of any material at a temperature *T* is governed by the dimensionless figure of merit *ZT* (*ZT* = *S*^*2*^*σT/κ*, where *S*, *σ* and *κ* denote the Seebeck coefficient, electrical conductivity and thermal conductivity, respectively [11, 12]. Reduced dimensionality of these materials has been proven to be one of the most common approaches to minimize the thermal conductivity and to obtain high *ZT* [13]. To minimize the thermal conductivity, it is very important to understand the phonon dynamics in this type of material, particularly the phonon–phonon and electron–phonon interactions, all of which have a great impact on the thermoelectric device performance [14, 15].

Raman scattering has been proved as an important tool for probing the vibrational modes in a material based on its non-destructive and microscopic nature [16, 17]. It also provides important information on doping, strain engineering and crystal phases [18, 19]. While the room-temperature Raman characterizations of phonon modes in various 2D TIs have been well studied in the literature [20, 21], temperature dependence Raman characterizations are still in the nascent stage. Furthermore, it is well known that change of temperature can vary inter-atomic distances and affect various phonon modes in the crystal [14]. Therefore, temperature-dependent Raman spectra are well suitable to obtain information on the thermal conductivity of materials, as well as isotopic effects and phonon lifetimes [22, 23].

In this work, we present power-dependent Raman spectroscopy at room temperature and temperature-dependent Raman spectroscopy in the temperature range between 100 and 300 K of 2D Sb_2_Te_3_ crystals of various thicknesses. The variation of Raman peak position and full width at half maximum (*FWHM*) with respect to temperature and power have been analyzed, and the results are interpreted to determine the thermal expansion coefficient and thermal conductivity of Sb_2_Te_3_ flakes in the context of thermometry study. The value of thermal conductivity for Sb_2_Te_3_ flake with a thickness of 115 nm has been estimated, and the role of the substrate to enhance the thermal conductivity has been discussed.

## Methods

Mechanical exfoliation was carried out on high-quality bulk Sb_2_Te_3_ crystal (2D Semiconductors, USA) using standard scotch tape technique [24] to obtain Sb_2_Te_3_ flakes of different thickness (65 nm, 80 nm, 115 nm, 200 nm and 400 nm) on 300-nm SiO_2_/Si substrates. Exfoliated samples were identified with the help of an optical microscope (LV100ND- Nikon Microscope). The lateral sizes of the Sb_2_Te_3_ nanoflakes are found in the range of 5–7 μm. Park NX-10 AFM (atomic force microscopy) was used to measure the thickness of the Sb_2_Te_3_ flakes using non-contact mode.

The Raman spectra were measured on various flakes using a HORIBA LabRAM confocal micro-Raman system in a backscattering geometry using a 632-nm laser excitation. A laser with spot size ~ 1 µm and tunable optical power from ~ 0.4 to 2.6 mW was used as the excitation source. The spectra were collected using a spectrometer equipped with a liquid-nitrogen-cooled CCD camera. The spectra were acquired in the frequency range from 100 to 200 cm^−1^ with a spectral resolution of 1 cm^−1^. All the measurements were taken using an integration time of 10 s, acquisitions of 10, and 1800 grating. For room temperature (RT) measurements, 100 × objective was used, while long working distance 50 × objective was used for low-temperature measurements.

## Results and Discussion

**Fig. 1:**
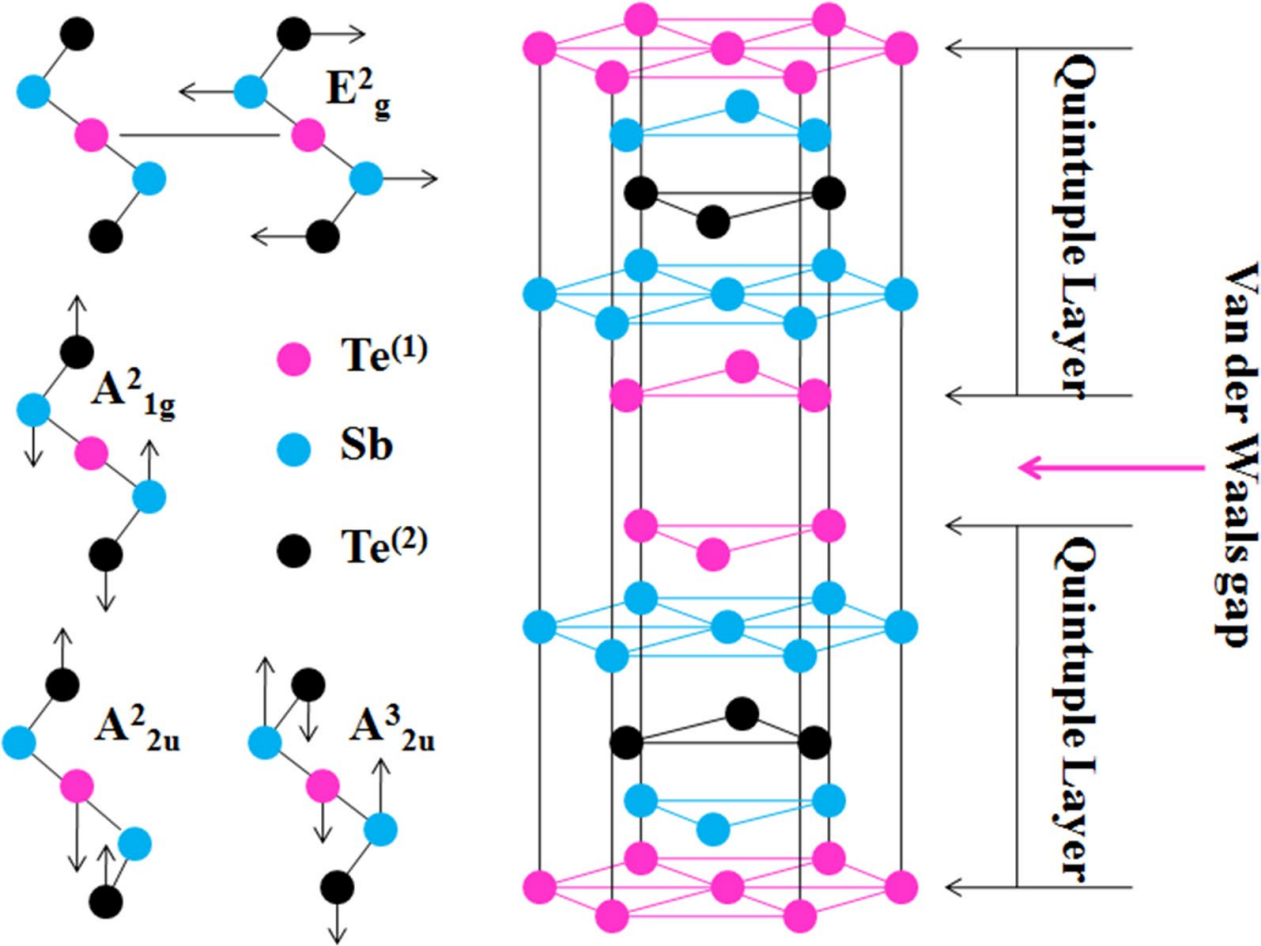
Schematic of Sb_2_Te_3_ crystal showing the arrangement of atoms and van der Waals gap. The pink, light blue and black circles represent the Te^(1)^, Sb and Te^(2)^ atoms, respectively. The left panel shows the possible phonon modes in the frequency range 100 cm^−1^ to 200 cm^−1^. The arrows represent direction of vibrations of constituent atoms

Sb_2_Te_3_ is a TI, which crystallizes in the rhombohedral crystal structure with space group *D*^5^_3*d*_($R\overline{3}m$), and its unit cell contains five atoms [20]. This crystal is formed by stacking five-atom layers along the *z-*direction, which is known as a quintuple layer (QL) as shown in Fig. 1, with a thickness of about 0.96 nm [20]. From the atomic registry, we can see that the Sb atom is sandwiched between two Te atoms, with the Te^(2)^ atom acting as an inversion centre. This centrosymmetric property of the crystal structure gives rise to mutually independent Raman active modes. The atoms within a single QL are held together by strong covalent forces, while the force between QLs is much weaker and of van der Waal’s type. Due to weak van der Waal force in the out-of-plane direction, it is possible to mechanically exfoliate thin layers of this material from its bulk crystals. Although exfoliated samples retain the composition and structure of bulk crystals, there is a change in phonon dynamics, when its thickness is reduced to the nanoscale level [25, 26].

**Fig. 2:**
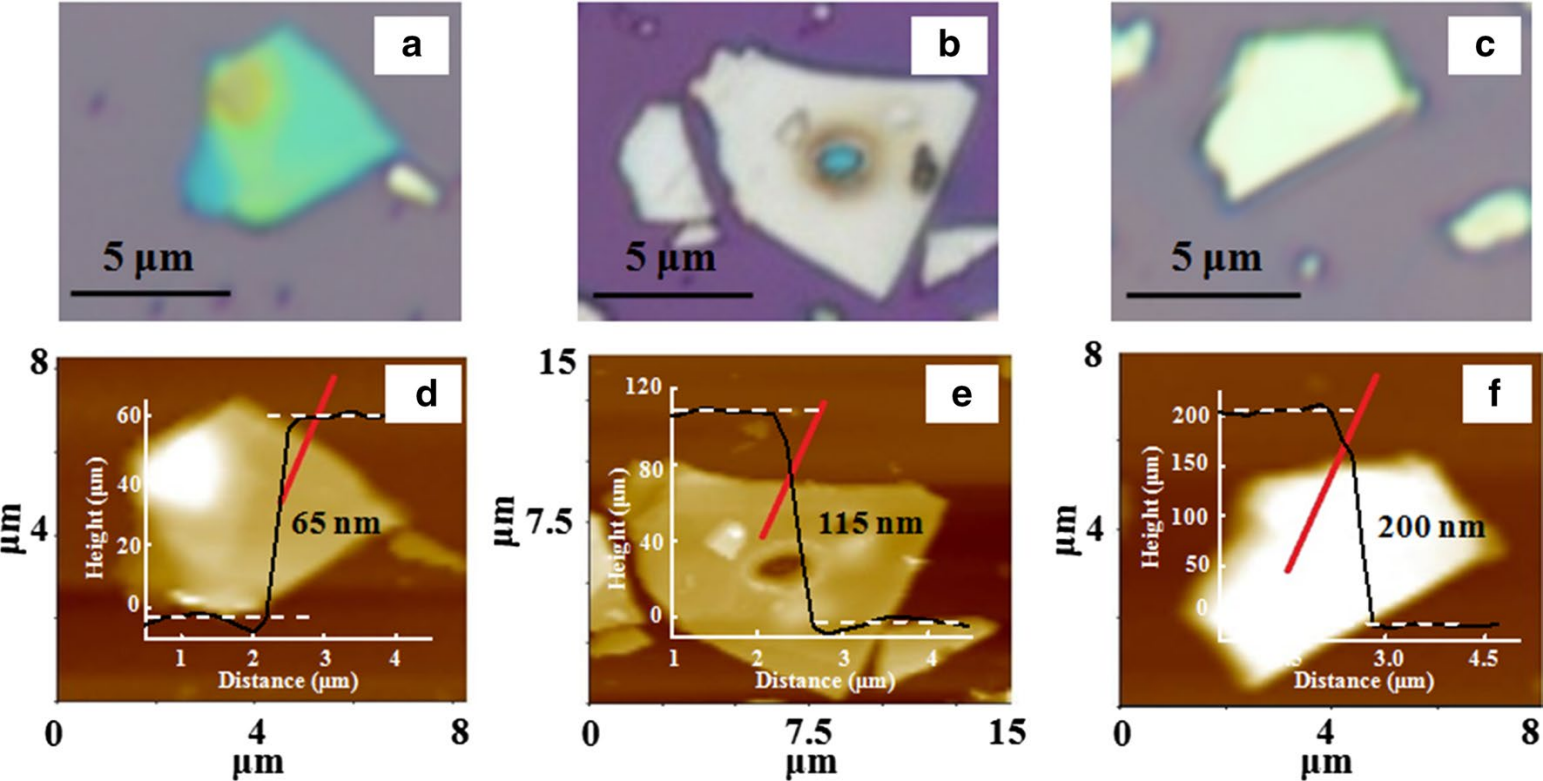
** a-c** OM images of Sb_2_Te_3_ flakes of thicknesses of 65 nm, 115 nm and 200 nm, respectively. **d-f** Their representative AFM images and height profiles.

Optical micrograph (OM) images of three different Sb_2_Te_3_ nanoflakes exfoliated on SiO_2_/Si substrate are shown in Fig. 2a-c. The lateral sizes of the flakes are in the range of 5–7 μm, which are large enough to be observed in OM. One can observe that the color contrast of the flakes is very sensitive to the thickness of the flakes *i.e.,* different thicknesses show different color contrast. The thicknesses of these prepared flakes were measured by atomic force microscopy (AFM), which are displayed in the lower panel of Fig. 2 along with their cross-sectional height profiles (Fig. 2d–f). The thickness values of these flakes were estimated to be 65 nm, 115 nm and 200 nm and were found to be almost uniform except for some bumps. But, all the Raman measurements were taken on the position of the flakes, where uniformity was maintained.

**Fig. 3:**
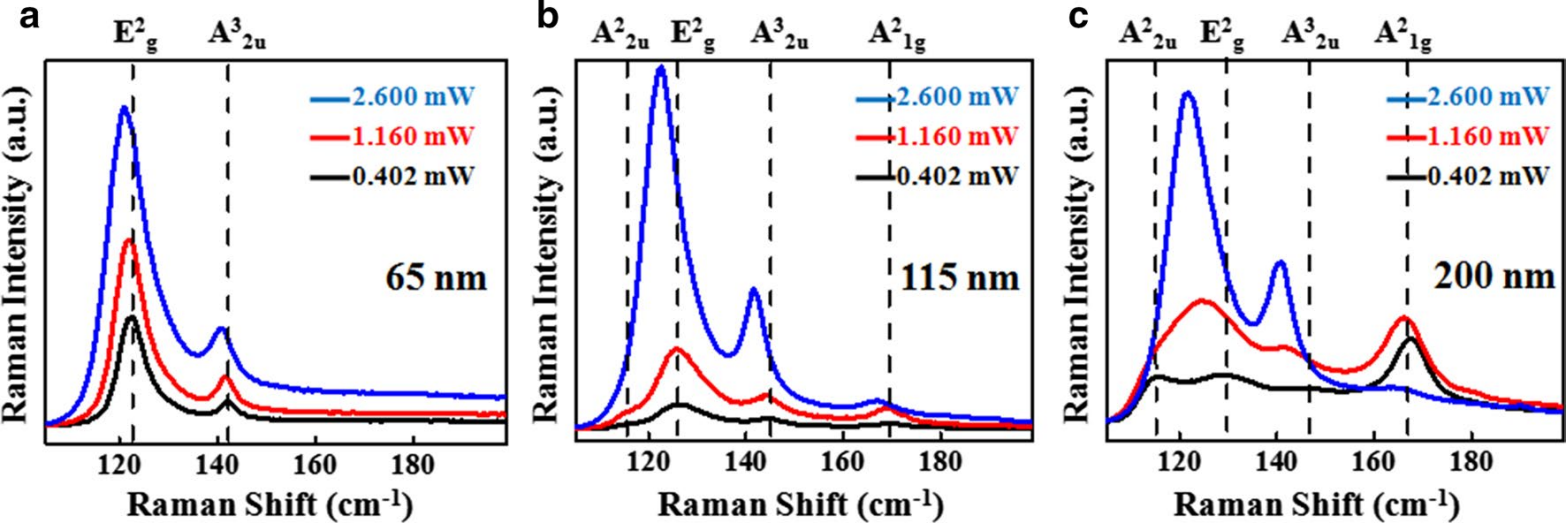
**a-c** Power-dependent micro-Raman spectra of 65-nm, 115-nm and 200-nm Sb_2_Te_3_flakes, respectively. The spectra are measured using 632-nm laser with three different powers 0.402 mW, 1.160 mW and 2.600 mW. The dashed lines show the position of the Raman modes.

Figure 3 presents the power-dependent Raman spectra of above three flakes measured at room temperature, which consists of four vibrational modes including two Raman active modes E^2^_g_ and A^2^_1g_ assigned at frequencies ~ 125 cm^−1^ and ~ 169 cm^−1^, and two IR active modes A^2^_2u_ and A^3^_2u_ assigned at ~ 115 cm^−1^ and ~ 144 cm^−1^, respectively [20, 27]. It is clearly observed that there is a red shift as well as an increase in the peak intensity of all the Raman modes with increase in laser power for all the flakes (65 nm, 115 nm and 200 nm). These changes suggest that the increase in laser power leads to a considerable increase in the local temperature on the surface of the sample [28]. Sb_2_Te_3_ flakes with the thicknesses of 115 nm and 200 nm exhibit all the four modes (A^2^_2u_, E^2^_g_, A^3^_2u_ and A^2^_1g_) for low laser power of 0.402 mW, and A^2^_2u_ and E^2^_g_ modes are merged together with further increase in power, which can be seen from the asymmetric line width of A^2^_2u_/E^2^_g_ modes in Fig. 3b, c. Figure 3a shows Raman spectra of Sb_2_Te_3_ flake with a thickness of 65 nm at three different incident laser powers, and the entire spectra exhibit only two Raman modes E^2^_g_ and A^3^_2u_ at room temperature. In this case, the shape of E^2^_g_ peaks for all laser powers looks asymmetry, which implies that there is also merging of both A^2^_2u_ and E^2^_g_ modes similar to thick flakes (115 nm, 200 nm) at high laser power. However, the A^2^_1g_ is completely absent for this thickness. We believe that this mode would be the characteristics of out-of-plane vibration, which would not be so significant for this thickness.

**Fig. 4:**
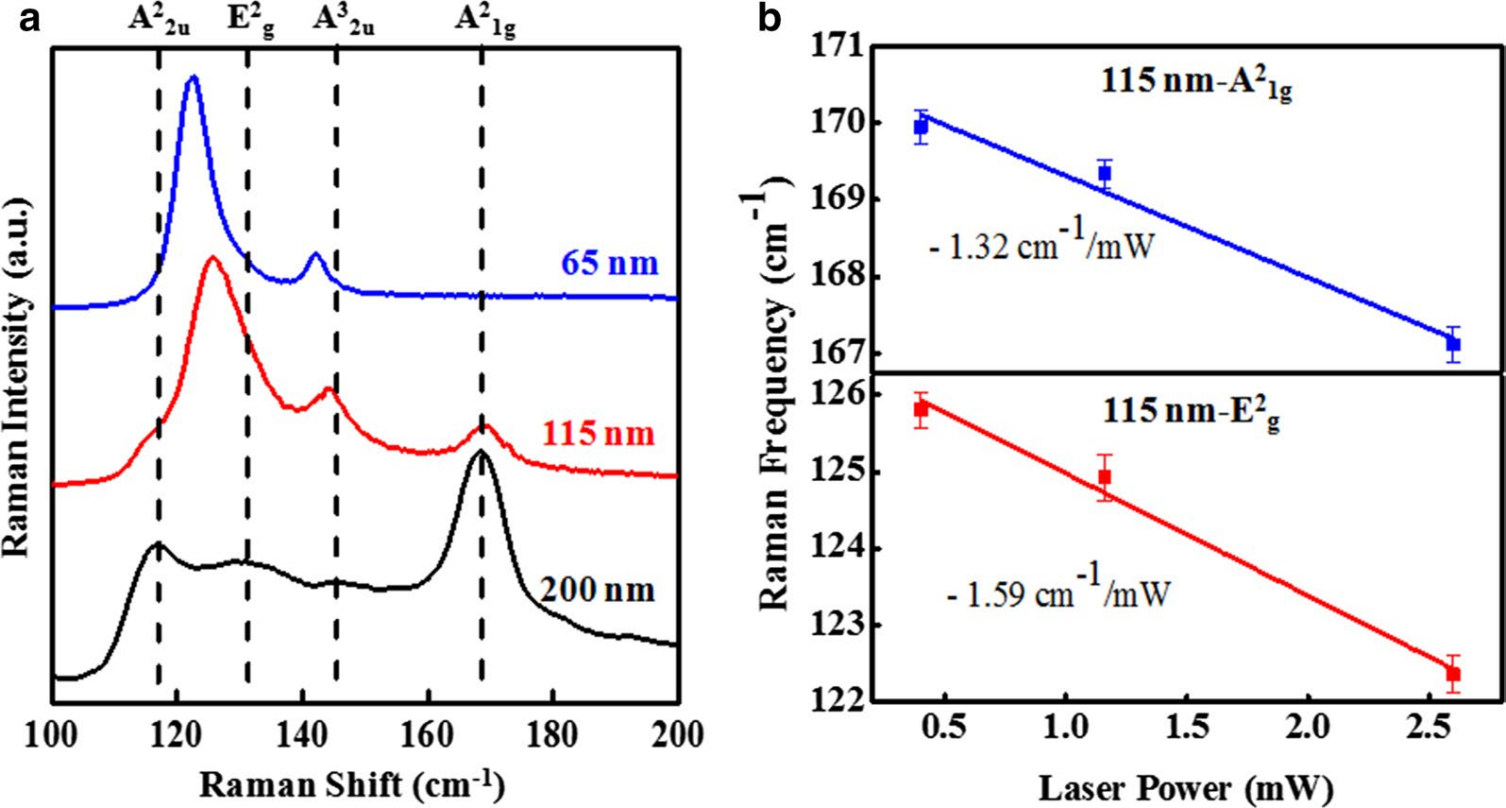
**a** Comparison of thickness-dependent micro-Raman spectra of 65-nm, 115-nm and 200-nm Sb_2_Te_3_ flakes at 0.402 mW laser power. The dashed lines show the position of the Raman modes. **b** Raman frequency vs. laser power plots of E^2^_g_ & A^2^_1g_ modes for 115-nm Sb_2_Te_3_ flake. The solid lines are the linear fits to the experimental data (symbols). The calculated slopes from the linear fits are shown as insets. The uncertainty in the Raman frequency measurement has been shown as error bars

The Raman spectra comparison of three different thicknesses (65 nm, 115 nm and 200 nm) samples at a particular laser power 0.402 mW is presented in Fig. 4a. All the observed Raman modes and their assignments are listed in Table 1. It is very interesting to observe that A^2^_1g_ and A^2^_2u_ modes for 200-nm flake possess more intensity than the other two modes (E^2^_g_ and A^3^_2u_). A^2^_1g_ and A^2^_2u_ modes are more sensitive to the thickness because it reflects out-of-plane vibrations and the inter layer van der Walls interactions. In the case of Sb_2_Te_3_ flakes with the thickness of 65 nm and 115 nm, the shape of E^2^_g_ peaks for all laser powers looks asymmetry, which implies that there is a merging of both A^2^_2u_ and E^2^_g_ modes. However, the A^2^_1g_ is completely absent for Sb_2_Te_3_ flake with the thickness of 65 nm. This particular Raman mode would originate due to out-of-plane vibration, which might be unresponsive for this thickness. A red shift is observed for E^2^_g_ and A^3^_2u_ phonon modes in the case of thinner flakes, similar to that reported by Zang et al. [30], whereas A^2^_1g_ mode shows a slightly blue shift (see Table 1). The peak intensities of 65-nm Sb_2_Te_3_ flake are found to be more pronounced than thicker ones under the same excitation laser power, and this phenomenon can be attributed to optical interference enhancements occurring for both the excitation laser and the emitted Raman radiation in the layered TI/SiO_2_/Si system [30], which is also reported for Bi_2_Se_3_ and Bi_2_Te_3_ [26, 31]. From the power-dependent Raman spectra of 115-nm Sb_2_Te_3_ flake (Fig. 3b), the Raman frequencies of E^2^_g_ & A^2^_1g_ modes have been extracted as a function of laser power as shown in Fig. 4b. The change in the phonon frequency with change in the incident laser power *i.e.,* power coefficient (*δω/δP*) has been estimated from linear fit to extracted data, which is found to be − 1.59 cm^−1^/mW and − 1.32 cm^−1^/mW corresponding to E^2^_g_ and A^2^_1g_ modes.

**Table1:** Positions of Raman modes (A^2^_2u_, E^2^_g_, A^2^_1g_ and A^3^_2u_) of three different Sb_2_Te_3_ flakes (65 nm, 115 nm and 200 nm) at room temperature with 0.402 mW laser power

	A^2^_2u_	E^2^_g_	A^3^_2u_	A^2^_1g_
65 nm	–	122.65	142.20	–
115 nm	115.23	125.91	144.57	169.17
200 nm	116.42	130.07	145.46	168.28

**Fig. 5:**
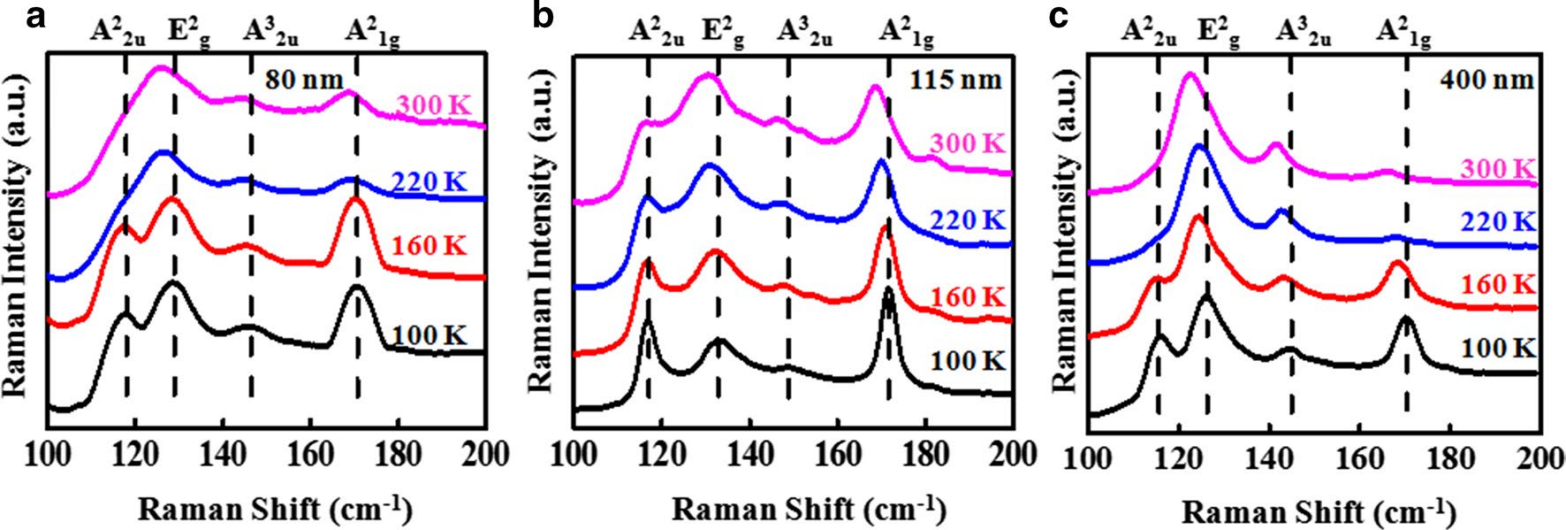
**a-c** Temperature-dependent micro-Raman spectra of Sb_2_Te_3_ of thickness 80 nm, 115 nm and 400 nm, respectively. The black-, red-, blue- and light blue-colored curves represent the Raman spectra at 100 K, 160 K, 220 K and 300 K, respectively, for 1.16 mW laser power. The dashed lines show the position of the Raman modes.

The temperature-dependent Raman spectra were measured in the temperature range from 100 to 300 K as shown in Fig. 5 for three different flakes with the thicknesses 80 nm, 115 nm and 400 nm, respectively, at 1.16 mW laser power. The OM, AFM images along with height profiles of 80-nm and 400-nm exfoliated Sb_2_Te_3_ flakes are given in Additional file 1: supporting information S1. At the lower temperature of 100 K, four characteristics Raman modes (A^2^_2u_, E^2^_g_, A^2^_1g_ and A^3^_2u_) of Sb_2_Te_3_ are clearly distinguishable, whereas A^2^_2u_ and E^2^_g_ Raman modes get merge together toward higher temperatures *i.e.,* 220 K and 300 K. Red shift and peak broadening were observed all the Raman modes (A^2^_2u_, E^2^_g_, A^2^_1g_ and A^3^_2u_) with the increase in temperature from 100 to 300 K. In general, temperature-dependent Raman spectroscopy is widely used to investigate the thermal expansion, thermal conduction and interlayer coupling [15, 31, 32]. In addition, the peak frequency has a linear dependence with the temperature, which is given by [15],

1 $$\omega \left( T \right) = \omega_{0} + \chi T$$

where *ω*_*0*_ is the frequency of vibration of these phonon modes at absolute zero temperature, and *χ* is the first-order temperature coefficient of these phonon modes. It has been reported that thermal expansion and contraction of the crystal and phonon modes may lead to the dependency of the peak position in Raman spectroscopy with temperature [33].

**Fig. 6:**
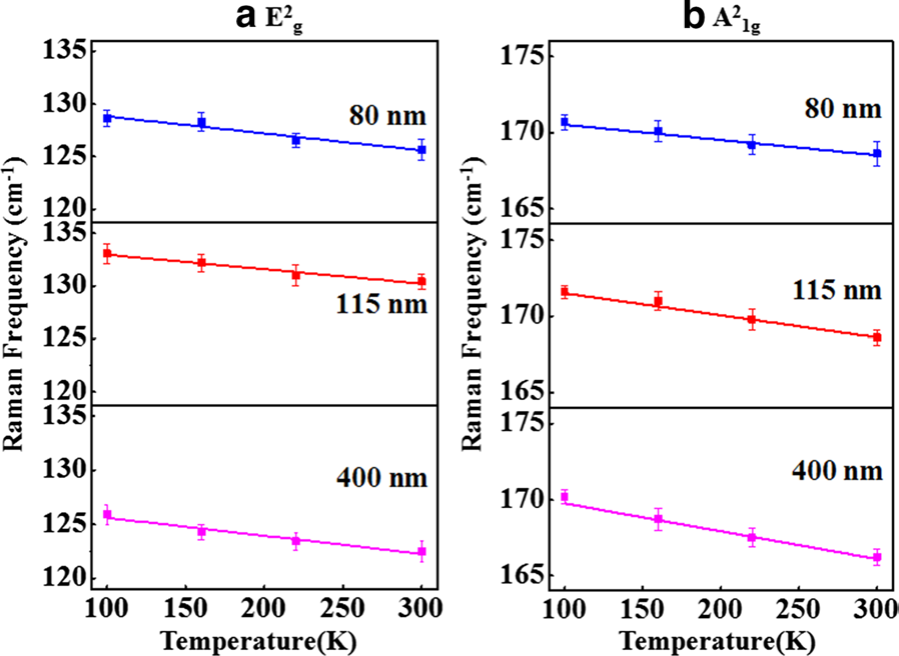
Raman frequency vs. temperature plots of **a** E^2^_g_ mode and **b** A^2^_1g_ mode for 80-nm, 115-nm and 400-nm Sb_2_Te_3_ flakes. The solid lines are the linear fits to the experimental data (symbols). The uncertainty in the Raman frequency measurement has been shown as error bars

**Fig. 7:**
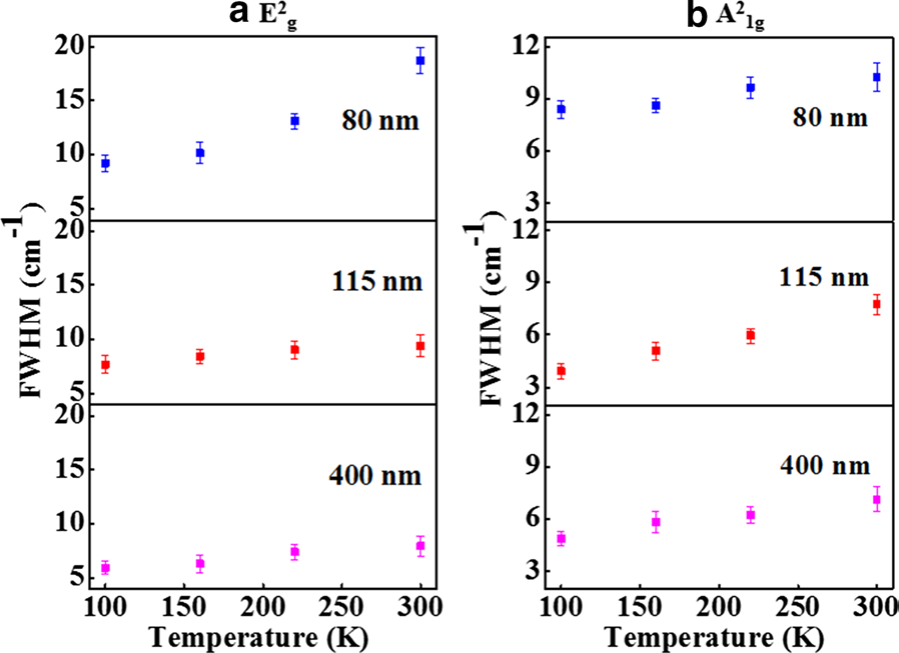
FWHM vs. temperature plots of **a** E^2^_g_ mode and **b** A^2^_1g_ mode for 80-nm, 115-nm and 400-nm Sb_2_Te_3_ flakes. The uncertainty in the FWHM estimation has been shown as error bars

The peak position versus temperature plots of E^2^_g_ & A^2^_1g_ modes are shown in Fig. 6a, b, respectively, for different thickness samples. The peak position versus temperature plots (Fig. 6a, b) have been linearly fitted using Eq. 1 to calculate the first-order temperature coefficient (*χ*), and the values of first-order temperature coefficient for E^2^_g_ & A^2^_1g_ Raman modes are listed in Table 2. The broadening in *FWHMs* of E^2^_g_ & A^2^_1g_ Raman modes with increase of the temperature is shown in Fig. 7a, b, respectively. The temperature dependence of the *FWHM* is a measure of phonon anharmonicity, and it increases linearly with increase in temperature. The simplest anharmonic approximation, known as the symmetrical three phonon coupling model [34], takes into account the optical phonon decay into two phonons with equal energies and opposite momenta. In the present work, we have calculated the first-order temperature coefficient (*χ)* and thermal conductivity from temperature-dependent Raman spectra. However, we are not analyzing the *FWHM* in the context of *ZT* as it has no such direct relevance to it.

**Table 2:** The first-order temperature coefficient (χ) for different Raman modes of some 2D thermoelectric materials along with the present work

Material	Temperature coefficient (cm^−1^/K)			Ref
TMDCs	χ- E^1^_**2g**_	χ- A_1g_		
MoS_2_ (single layer)	− 0.013	− 0.016		[35]
MoS_2_ (few layer)	− 0.016	− 0.011		[36]
WSe_2_ (single layer)	− 0.0048	− 0.0032		[37]
Trichalcogenides		χ-A_1g_	χ-A_2g_	
TiS_3_ nanoribbons		− 0.018	− 0.021	[38]
TiS_3_ nanosheets		− 0.02	− 0.024	[38]
TIs	χ-A^1^_1g_	χ-E^2^_g_	χ-A^2^_1g_	
Bi_2_Se_3_ nanoplates	− 0.01258	− 0.01385	− 0.02363	[15]
Bi_2_Te_3_ nanowires			− 0.0128	[39]
Sb_2_Te_3_ (400 nm)		− 0.016	− 0.018	This work
Sb_2_Te_3_ (115 nm)		− 0.014	− 0.015	This work
Sb_2_Te_3_ (80 nm)		− 0.016	− 0.01	This work

It is observed that the value of first-order temperature coefficients (*χ*) for E^2^_g_ and A^2^_1g_ mode is in order of 10^–2^ cm^−1^/K. The value of *χ* corresponding to A^2^_1g_ mode decreases from − 2 × 10^–2^ to − 1 × 10^–2^ cm^−1^/K when the thickness of Sb_2_Te_3_ flake is reduced from 400 to 80 nm. Such low *χ* would give low thermal conductivity and favor to get a high figure of merit (*ZT*). However, the value of *χ* corresponding to E^2^_g_ mode is almost constant and is independent of thickness. Now, we have calculated an approximate value of thermal conductivity of Sb_2_Te_3_ flake using the power coefficient and first-order temperature coefficient values. The heat conduction through a surface with the cross-sectional area *S* can be evaluated from the following equation: $\partial Q/\partial t = -\kappa{\oint }\nabla T.dS,$ where *Q* is the amount of heat transferred over the time *t* and *T* is the absolute temperature. Considering the radial heat flow, Balandin et al*.* [40] have derived an expression for thermal conductivity of graphene, which is given by

2 $$= \left( {1/2\pi h} \right)\left( {\Delta P/\Delta T} \right)$$

where *h* is the thickness of the 2D film of the material and the local temperature rise *ΔT* is due to the change in heating power *ΔP*. By differentiating Eq. (1) with respect to power and substituting (*ΔP/ΔT*) in expression (2), the thermal conductivity can be written as follows,

3 $$= \chi \left( {\frac{1}{2\pi h}} \right)\left( {\frac{\delta \omega }{{\delta P}}} \right)^{ - 1}$$

where *κ* is thermal conductivity, *h* is the thickness of the 2D film of the material, *χ* is the first-order temperature coefficient, and (*δω/δP*) is change in the phonon frequency with change in the incident laser power *i.e.,* power coefficient of particular Raman modes. The calculated thermal conductivity is found to be ~ 10 W/m–K for Sb_2_Te_3_ flake with the thickness of 115 nm supported on 300-nm SiO_2_/ Si substrate. This value is relatively higher than the reported thermal conductivity of other TI [41]. The slight enhancement in thermal conductivity suggests that the supporting substrate plays a more sensitive role *i.e.,* the value of thermal conductivity might be dependent on interfacial charges [42]. This higher thermal conductivity at the substrate-supported sample can also explain the smaller temperature rise under high laser power in comparison with the suspended sample. The similar substrate effect is also reported in Su et al*.* for black phosphorus layers [42]. Guo et al*.* also reported that, in certain regions, the effect of phonon scattering can be suppressed and the thermal conductivity of nanomaterials can be surprisingly increased due to the coupling induced shift of phonon band to the low wave vector [43]. Recently, a theoretical study on the substrate effect of the thermal conductivity of graphene has been also reported. The authors also found that both the reduction and the increment of thermal conductivity can be induced by the substrate, depending on the coupling condition [44]. From Eq. 3, thermal conductivity is directly proportional to the first-order temperature coefficient, and it is well known that figure of merit (*ZT*) is inversely proportional to the thermal conductivity. Hence, low *χ* and *κ* are promising to achieve high *ZT*.

Further work is in the process to achieve Sb_2_Te_3_ nanoflake with thickness less than 7 QL, which is the confinement limit of 2D TI using exfoliation technique with help of special-type scotch tape or by using chemical vapor deposition. Such low thickness flakes are expected to yield a very low-temperature coefficient (~ 10^–3^ to 10^–4^ cm^−1^/K) and a high *ZT*. With high *ZT*, 2D Sb_2_Te_3_ would have great potential in the field of thermoelectric applications.

## Conclusions

In conclusion, we have successfully synthesized 2D Sb_2_Te_3_ of various thicknesses in the range of 65–400 nm using mechanical exfoliation and studied the thermometry of these nanoflakes. The temperature dependence of the peak position and line width of phonon modes A^2^_1g_ and E^2^_g_ modes were analyzed to determine the temperature coefficient, which is found to be in the order of 10^–2^ cm^−1^/K. The temperature coefficient in the out-of-plane direction decreases with decrease in Sb_2_Te_3_ thickness. Such a low-temperature coefficient would favor to achieve a high *ZT* and pave the way to use this material as excellent candidates of thermoelectric materials. Using temperature coefficient and power coefficient values, the thermal conductivity of 115-nm Sb_2_Te_3_ flake supported on 300-nm SiO_2_/ Si substrate was estimated to be ~ 10 W/m–K. The slightly higher thermal conductivity compared to other TIs suggests that the supporting substrate significantly affects the heat dissipation of the Sb_2_Te_3_ flake.

## Supplementary information

**Additional file 1.** The supplementary material contains the optical images & AFM images along with heightprofiles of 80-nm and 400-nm exfoliated Sb2Te3 flakes and Raman peak of silicon at 520.74cm-1.

## Data Availability

The data that support the findings of this study are available from the corresponding author upon reasonable request.

## Abbreviations

TIs: Topological insulators
*ZT*: Figure of merit
OM: Optical micrograph
AFM: Atomic force microscopy
*FWHM*: Full width at half maximum
QL: Quintuple layer
