# Supplementary material for: Study of Thermometry in Two-Dimensional Sb2Te3 from Temperature-Dependent Raman Spectroscopy
Source: Nanoscale Res Lett. 2021 Feb 3;16:22. doi: 10.1186/s11671-020-03463-1 (PMC7859149; doi:10.1186/s11671-020-03463-1)
Supplement: Supplementary file 1 — Additional file 1. The supplementary material contains the optical images & AFM images along with heightprofiles of 80-nm and 400-nm exfoliated Sb2Te3 flakes and Raman peak of silicon at 520.74cm-1. [file 11671_2020_3463_MOESM1_ESM.docx]

***Supporting Information for***

**Study of thermometry in two-dimensional Sb_2_Te_3_ from temperature dependent Raman spectroscopy**

Manavendra P. Singh^1†^, Manab Mandal^2†^, K. Sethupathi^2^, M.S. Ramachandra Rao^1,3^, Pramoda K. Nayak^1^*

^1^Department of Physics and Materials Science Research Centre, Indian Institute of Technology Madras, Chennai 600 036, India

^2^Department of Physics, Indian Institute of Technology Madras, Chennai 600 036, India

^3^Nano Functional Materials Technology Centre, Indian Institute of Technology Madras, Chennai 600 036, India

†These authors contributed equally to this work

Corresponding author; **Email:* [pnayak@iitm.ac.in](mailto:pnayak@iitm.ac.in)

**
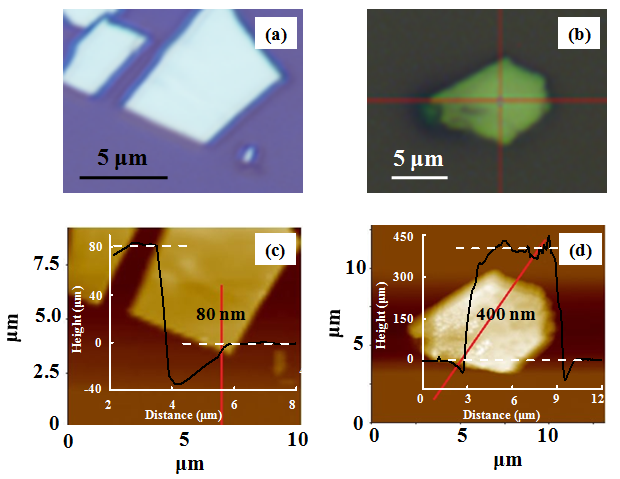
**

**Figure S1.** (a, b) OM images of mechanically exfoliated Sb_2_Te_3_ flakes of thicknesses of 80 nm and 400 nm respectively. (c,d) Their representative AFM images and height profiles.


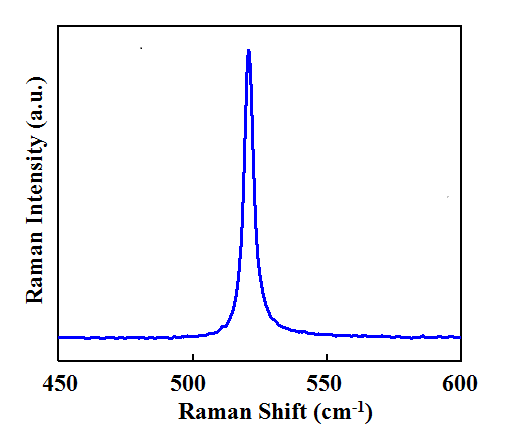


**Figure S2.** Raman peak of Silicon at 520.74 cm^-1^.
